# Semisynthesis, an Anti-Inflammatory Effect of Derivatives of 1β-Hydroxy Alantolactone from *Inula britannica*

**DOI:** 10.3390/molecules22111835

**Published:** 2017-10-27

**Authors:** Lin Chen, Jian-Ping Zhang, Xin Liu, Jiang-Jiang Tang, Ping Xiang, Xing-Ming Ma

**Affiliations:** 1 Department of Immunology, School of Basic Medical Sciences, Lanzhou University, Lanzhou 730000, China; chenlinfree@126.com; 2 Department of Infectious Disease, the First Hospital of Lanzhou University, Lanzhou 730000, China; liuxin1288@126.com; 3 Department of Pharmacy, the First Hospital of Lanzhou University, Lanzhou 730000, China; Zhangjianping0306@163.com; 4 College of Chemistry & Pharmacy, Northwest A&F University, Yangling 712100, China; tangjiang11@nwafu.edu.cn; 5 College of Plant Protection, Northwest A&F University, Yangling 712100, China

**Keywords:** 1β-hydroxy alantolactone, semisynthesis, anti-inflammatory activity

## Abstract

1β-hydroxy alantolactone, a sesquiterpene lactone mainly isolated from *Inula* genus plants, exhibits potent anti-inflammatory and anticancer activities. In this work, 1β-hydroxy alantolactone was isolated and five derivatives were prepared through different reactions at the C1-OH and C13-methylene motifs. The structure–activity relationships (SAR) of anti-inflammatory effects against NO production in RAW264.7 cells showed that the α-methylene-γ-butyrolactone motif was essential for NO production suppression and that retaining the C1-OH group can remarkably improve this effect. The NF-κB signaling pathway plays a pivotal role in the regulation of NO expression. Moreover, the levels of p65 and p50 phosphorylation were investigated and active compound **1** inhibited phosphorylation of p65 and p50 in TNF-α-induced NF-κB signaling. Further molecular docking suggested that **1** may target the p65 of NF-κB.

## 1. Introduction

**Figure 1:**
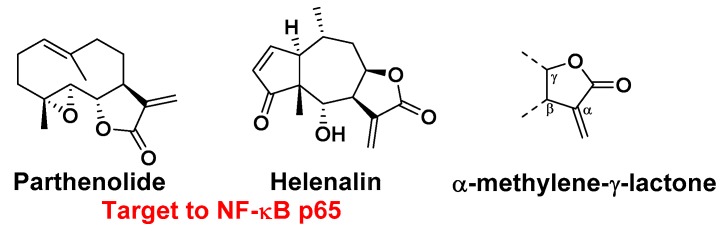
Chemical structure of representative anti-inflammatory sesquiterpene lactones (STLs) and relevant active moieties.

Naturally-occurring sesquiterpene lactones (STLs) are plant-derived bioactive constituents often used in traditional medicines against inflammation, cancer, malaria, and viral and bacterial infection [1,2,3,4,5,6,7]. There are about 1500 publications reporting on the anticancer and anti-inflammatory properties of STLs [3,8,9], in which parthenolide and helenalin are representative STLs of particular significance (Figure 1). Parthenolide (with a 10-membered ring), isolated from the medicinal herb feverfew, is now in cancer clinical trials due to its potential anti-inflammatory, anticancer, and antiviral properties [3]. Helenalin (a 5/7-fused bicyclic system) found in various Asteraceae such as *Arnica* spp., could inhibit the activity of the telomerase, therefore being a potential anti-cancer agent [10]. The mechanisms of action of parthenolide and helenalin on cell functionality were investigated and found to inhibit transcription factor NF-κB, resulting in decreased recruitment of the T- and B-cell signaling pathway [11].

The canonical NF-κB signaling pathway is a mediator of the cellular inflammatory response, and aberrant activation of NF-κB is perhaps implicated in a spectrum of human diseases, including chronic inflammatory disease, atherosclerosis, and cancer [12]. One of these STLs structural features is the α-methylene-γ-butyrolactone moiety (Figure 1), which allows STLs to react with proteins via the Michael addition, especially thiol groups of some proteins, resulting in their alkylation. Moreover, both STLs (parthenolide and helenalin) were previously shown to target Cys38 in the p65 of NF-κB and ablate its DNA-binding ability [13,14].

**Figure 2:**
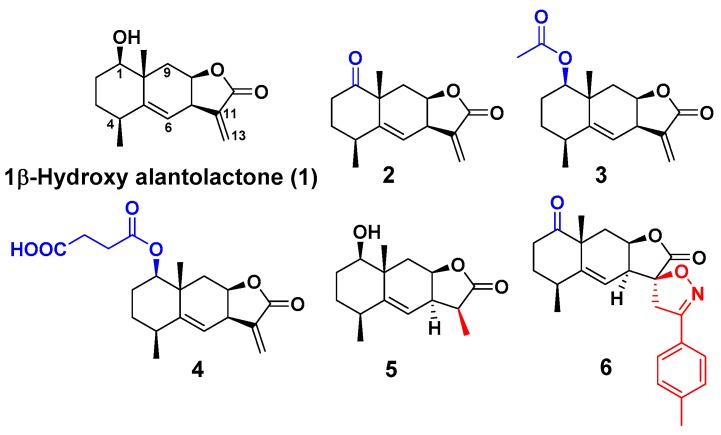
Semisynthetic derivatives of 1β-hydroxy alantolactone (**1**).

1β-Hydroxy alantolactone (**1**, a 6/6-fused bicyclic system, Figure 2), mainly isolated from the *Inula* genus (*Inula britannica*, *Inula japonica* and *Inula helenium*) plants [1,15,16], have shown anticancer and anti-inflammatory effects [1,17]. α-methylene-γ-butyrolactone has been considered as its active moiety. However, to our best knowledge, an anti-inflammatory structure–activity relationship (SAR) for **1** has been not reported with respect to whether or not other functional groups inflect its biological activity, and its further mode of action remains unknown. In our ongoing efforts in exploring SARs in anti-inflammatory potency, we herein semisynthesized five derivatives (**2**–**6**, Figure 2) including oxidized **2** and esterified **3** (with increased lipophilicity) and **4** (with increased hydrophilicity) at the C1-OH position, which reduced **5** and spiral **6** at the C13-methylene motif. Therein, specific spiro-cycle natural products have been reported as having potent biological activities [18,19,20,21,22], for example antitumor activities (artemalogues) [20] and induction of autophagy activity (clionamine D) [22]. Furthermore, their anti-inflammatory activities, including the inhibitory against lipopolysaccharide (LPS)-induced NO production and TNF-α-induced NF-κB signaling in RAW 264.7 macrophages were evaluated.

## 2. Results and Discussion

**Scheme 1:**
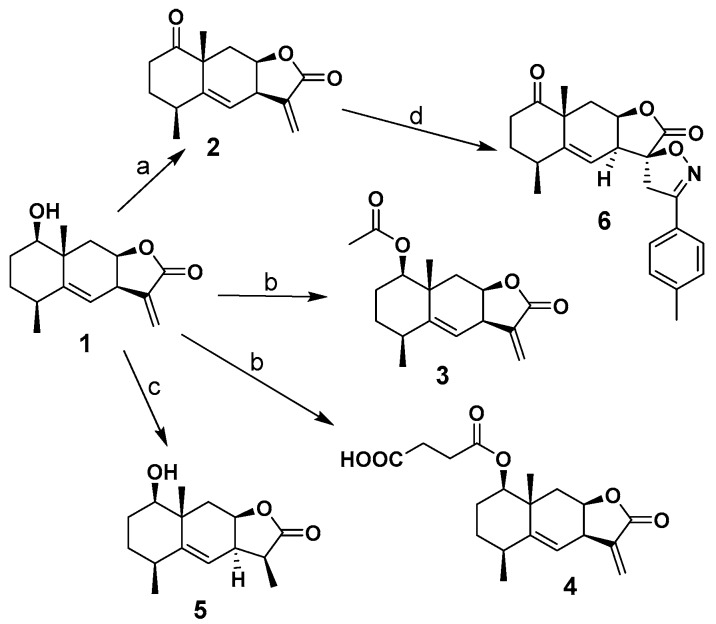
Semisynthetic route of 1β-hydroxy alantolactone (**1**) derivatives. Conditions and reagents: (**a**) Dess–Martin periodinane (DMP), CH_2_Cl_2_, r.t., 85%; (**b**) a different anhydride, Et_3_N, DMAP, r.t., 76% for **3** and 85% for **4**; (**c**) NaBH_4_, THF, 2 h, r.t., 67%; (d) (i) *p*-methylbenzaldehyde and 50% NH_2_OH in H_2_O, Et_2_O, 89%; (ii) DMF, NCS, r.t., 98%; (iii) CH_2_Cl_2_, Et_3_N, r.t., 76%.

1β-Hydroxy alantolactone (**1**) was firstly isolated from the dried flowers of *I. britannica* through repeated column chromatography in previous papers by the authors of this paper as well as others [1,17,23]. Then, **1,** as a starting material, was converted to the corresponding derivatives **2**–**6** by various reactions such as oxidation for **2**, esterification for **3** and **4** at C1-OH position, reduction for **5**, and 1,3-dipolar cycloaddition for **6** at C13-methylene (Scheme 1). The structures of all compounds were well characterized by NMR, (HR)ESI-MS and HPLC purity analysis referred to in the Supplementary Material. 

The potent anti-inflammatory properties of STLs have been reported to be chemically mediated by α-methylene-γ-butyrolactone and other functional groups such as the α,β-unsaturated carbonyl motif [24]. To investigate the SAR of 1β-hydroxy alantolactone, we investigated the anti-inflammatory effects of **1**–**6** through inhibition of the LPS-induced NO production in RAW 264.7 macrophages. Aminoguanidine (AG) was used as the positive control. Meanwhile, a cytotoxicity test was performed at the same time in order to explore the influence of cytotoxicity on the inhibition of NO production assay. The IC_50_ values of NO production suppression and the cytotoxicity of **1**–**6** in LPS-induced RAW 264.7 macrophages are summarized in Table 1. In the compound treatment periods, **1**–**4** retaining α-methylene moiety exhibited inhibitory effects against NO production with IC_50_ values of 5.61, 36.1, 46.5, and 39.6 μM, respectively. However, **5** and **6** with C13 reduction and cycloaddition showed no inhibitory activity (>1000 μM), suggesting that α-methylene-γ-butyrolactone is essential to the anti-inflammatory potency, which is consistent with previous reports [3,8]. Among these tested compounds, **1** bearing the C1-OH group displayed the highest potency against NO production, at a level 6.5–8.4 fold greater than that of **2**–**4**, and it is more active than aminoguanidine (positive control). This result hinted the importance of the C1-OH functionality in terms of anti-inflammatory potency.

In cytotoxicity test, **2** showed cytotoxicity with IC_50_ of 34.5 μM, yet other derivatives were not notably cytotoxic at the concentration (IC_50_ > 50 μM). However, precursor **1** exhibited stronger suppression of NO production (Table 1) but weak cytotoxicity (IC_50_ > 50 μM), indicating that C1-OH functionality is different between the cytotoxicity and anti-inflammatory effects.

**Figure 3:**
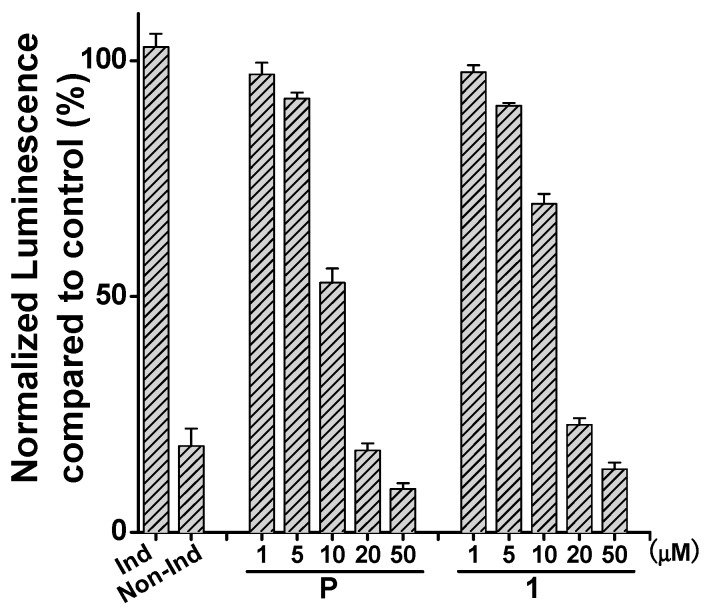
Compound **1** inhibited TNF-α-induced NF-κB activation by NF-κB-luciferase inhibition assay in RAW264.7 cells. **1** and parthenolide (**P**, positive control) were dosed to RAW264.7 cells containing a stably transfected NF-κB luciferase reporter and stimulated with TNF-α (50 ng/mL) for 8 h (except for a non-induced control, Non-Ind). Luminescence was normalized to the non-compound induced (Ind) control and plotted as NF-κB luciferase activity (%). Mean ± standard deviation values are shown with more than three replicates.

**Figure 4:**
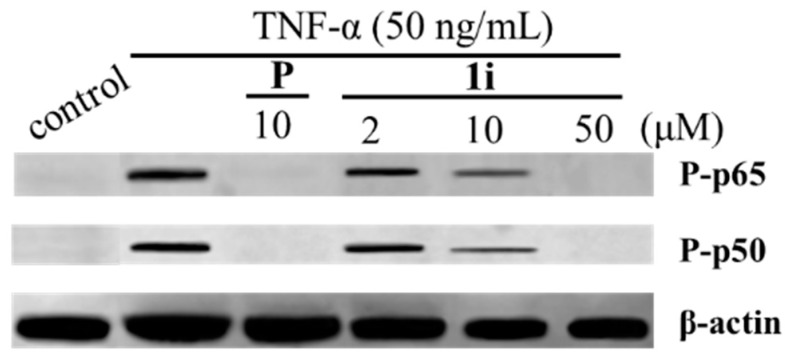
Compound **1** blocks TNF-α-induced NF-κB activation through p65 and p50 phosphorylation in RAW264.7 cells. After treatment with **1** and parthenolide (**P**) at the indicated concentrations for 8 h, total cell lysates were tested by western blot experiments.

NF-κB is a transcription factor that controls immune responses and plays a pivotal role in the regulation of NO expression [25]. In order to investigate the inflection of the active compound **1** on NF-κB signaling, we next conducted an NF-κB luciferase reporter assay in TNF-α-induced RAW264.7 cells to evaluate the impact of **1** on the transcriptional activity of NF-κB. Parthenolide (**P**) was utilized as a positive control (benchmark). It could be seen in Figure 3, after this 8 h treatment test, that **1** displayed dose-dependent inhibition (such as 75% inhibition at 20 μM) towards the NF-κB pathway. The observation that **1** and parthenolide were comparably potent in this assay implies the possibility that **1** may have similar properties, like parthenolide in cells. When treatment with compound **2** at the concentration of 20 μM, the NF-κB-luciferase inhibitory percentage was 55% (data not shown), which further indicated the importance of the C1-OH in NF-κB-luciferase inhibition in RAW264.7 cells. Moreover, the levels of p65 and p50 phosphorylation were investigated by western blotting analysis. As shown in Figure 4, **1** dose-dependently suppressed TNF-α-induced phosphorylation of the NF-κB p65 and p50 subunit, demonstrating inhibition potency of **1** in NF-κB signaling.

**Figure 5:**
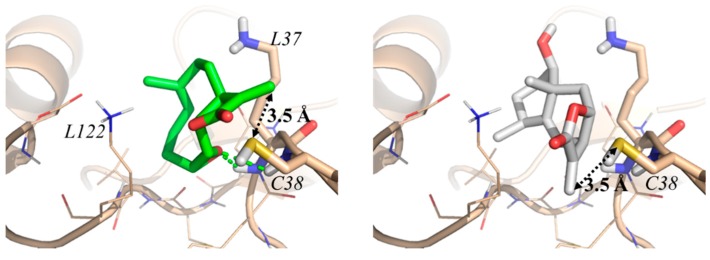
The docking mode of parthenolide (**P**, left, green) and **1** (right, gray) through binding to NF-κB/p65 (PDB: 1VKX). **1** can superimposed well with **P** in the p65 binding site and the spatial distance between both the α-methylene and SH group of C38 is about 3.5 Å.

The p65 of NF-κB is a main target of parthenolide [14]. To further understand the possible binding mode of **1** with p65, we simulated the molecular modeling of **1** and parthenolide (**P**) with p65 of NF-κB by the Surflex–Dock protocol using the Sybyl-X software package (PDB: 1VKX) [5,13]. The docking result (Figure 5) showed that **1** could superimpose well with parthenolide in a p65 binding site. The best matching interaction between **1** and p65 site exhibited that the spatial distance between the exocyclic methylene (C13) and -SH of Cys38 is about 3.5 Å, similar to that of parthenolide, implying that **1** may form covalent protein adducts with Cys38 of p65 [5].

## 3. Materials and Methods

### 3.1. General

Analytical HPLC was performed on a Waters 1525 series with an Agilent TC-C18 column and UV (PDA) detection at the max wavelength of compounds. Column chromatography (CC) was performed over silica gel (200–300 mesh, Qingdao Marine Chemical Ltd., Qingdao, China). The progress of all reactions was monitored by TLC on 2 cm × 5 cm precoated silica gel GF_254_ plates with a thickness of 0.25 mm (Qingdao Marine Chemical Group, Co., Qingdao, China). NMR spectra were recorded on a 500 MHz Bruker spectrometer. HRESI-MS spectra were obtained on a Thermo Scientific LTQ Orbitrap (Thermo Scientific, Waltham, MA, USA). Dulbecco’s modified Eagle’s medium (DMEM) and fetal bovine serum (FBS) were purchased from Invitrogen. Lipopolysaccharide (LPS) and Sulforhodamine B (SRB) were purchased from Sigma-Aldrich (St. Louis, MO, USA). A nitric oxide assay kit was purchased from Beyotime (Shanghai, China). All commercially available solvents and reagents were freshly purified and dried by standard techniques prior to use.

### 3.2. Extraction and Isolation from Plant Material

Air-dried flowers of *I. britannica* were extracted with 95% EtOH under reflux. 1β-hydroxy alantolactone (**1**) was isolated through repeated chromatography on a silica gel column according to a previous method and ^1^H, ^13^C-NMR, specific rotation, and ESI-MS were reported in our literature [17].

### 3.3. Synthesis of Derivatives

Using **1** as a starting material, derivative **2** was prepared through oxidation reaction with Dess–Martin periodinane (DMP) in 85% yield; **3** and **4** was synthesized by esterification reaction with acetic anhydride or succinic anhydride in 76% and 85% yield, respectively; **5** was obtained through NaBH_4_ reduction. Furthermore, **2** was converted to spiro[lactone-isoxazol] **6** with new-made aldoxime chloride by 1,3-dipolar cycloaddition reaction. Detailed semisynthetic methods and spectra data of **2**–**6** are referred to in the Supplementary Material.

### 3.4. Cell Culture

RAW 264.7 cell line was originally obtained from ATCC (American type culture collection). The cell line was grown in DMEM (Invitrogen, Carlsbad, CA, USA) containing 10% (*v*/*v*) thermally inactivated fetal bovine serum (FBS, Invitrogen), penicillin (100 KU/L) and streptomycin (100 KU/L) at 37 °C in a 5% CO_2_ humidified incubator.

### 3.5. Measurement of NO Production in RAW264.7 Macrophages

Then, 100-μL aliquots of the exponentially growing RAW 264.7 cells were seeded in 96-well culture plates at 1 × 10^4^ cells/well at 37 °C for overnight in DMEM medium with 0.1% FBS. The culture medium was replaced by fresh free-FBS medium and cells were pretreated with different concentrations (0, 1, 3, 10, 30, 100, 300, 1000 μM) of samples for 4 h. Thereafter, the medium with samples was removed and then incubated for 48 h with or without 2 μg/mL LPS 10% FBS DMEM. The nitrite concentration (NO production) in the culture supernatant was measured using Nitric Oxide assay kit mainly containing Griess reagent. The absorbance was measured at 540 nm using a microplate reader (M5). The amount of NO was calculated from a standard curve created using sodium nitrite with known concentrations. The cell viability was evaluated by SRB colorimetric assay [26].

### 3.6. Luciferase NF-κB Reporter Assay

RAW 264.7 cells (10,000 cells/well) were placed in a 96-well plate and the cells were then transfected with pNF-κB-Luc expression plasmid using a mixture of plasmid and lipofectamin 2000 PLUS in the Opti-MEM^®^ I Reduced Serum Medium according to manufacturer’s instructions (Life Technologies, Carlsbad, CA, USA). After 12 h, the cells were co-incubated with the active samples (1, 5, 10, 20, 50 µM) and TNF-α. Parthenolide (**P**) was used as positive control. NF-κB was induced by adding TNF-α (50 ng/mL, delivered in PBS) to the treated wells and the induced control wells. After an additional 8 h, Bright-Glo luciferase reagent (Beyotime) was added to each well (100 μL) and the plate was allowed to stand for two minutes. Luminescence measurements with a relative light unit (RLU) were then obtained using a Fluoroskan Ascent multiple microplate reader (Molecular Device, Sunnyvale, CA, USA). Each experiment was performed in biological triplicate (at minimum) with three technical replicates per experiment.

### 3.7. Western Blotting Analysis

RAW 264.7 cells were seeded in a 24-well plate at 5 × 10^4^ cells/well. After cells adherence for overnight, the test compound **1** (50, 10 and 1 µM) or parthenolide (10 µM) and TNF-α (50 ng/mL) were added to the wells and incubated for 8 h. Then, cell pellets were collected and lysed with RIPA lysis buffer containing 0.5 mM phenylmethylsulfonyl fluoride (PMSF) and a protease inhibitor cocktail. The protein concentration of cell samples was analyzed by using the BCA method. Equal amount proteins of each sample were electrophoresed on 12% separating gel and 4% stacking gel and electrotransferred onto an NC membrane. After incubation with appropriate primary and secondary antibodies of p65 and p50, protein blots were detected by using ECL solution and ChemiDoc XRS+ imaging system (Bio-Rad, Hercules, CA, USA). β-actin was used as loading control.

### 3.8. Molecular Modeling

Computational docking of the interaction of **1** with p65/NF-κB was performed with the Surflex–Dock protocol using Sybyl-X 2.1.1 software [27]. The original parameters of blind docking were used in combination with an evaluation scheme based on binding free energy (ΔG). The structure of NF-κB p50–p65 heterodimer bound to DNA was obtained from the Protein Data Bank (1VKX). 

### 3.9. Statistics Analysis

All the data were the mean ± standard deviation (SD). Statistical analysis was performed by the 2-tailed Student *t*-test using GraphPad software.

## 4. Conclusions

In conclusion, we semisynthesized five 1β-hydroxy alantolactone derivatives by four different reactions at the C1 and C13 positions and evaluated their anti-inflammatory effects in vitro. Among the derivatives, compound **1,** containing a C1-OH group, displayed significant inhibitory activity against NO production and a suppressive effect against TNF-α-induced NF-κB without strong cytotoxicity. Predicted binding modes showed that **1** may have the ability to form covalent adducts with Cys38 of p65. Further target identification will soon be underway.

## Figures and Tables

**Table 1:** Inhibitory activities against NO production and cytotoxicities of 1β-hydroxy alantolactone (**1**) and its derivatives in lipopolysaccharide (LPS)-induced RAW 264.7 macrophages.

Compound	IC_50_ (μM)	
	NO Inhibition ^1^	Cytotoxicity ^2^
**1**	5.61 ± 0.34	>50
**2**	36.1 ± 3.8	34.5 ± 5.8
**3**	46.5 ± 5.6	>50
**4**	39.6 ± 5.7	>50
**5**	>1000	ND ^4^
**6**	>1000	ND
Aminoguanidine	10.9 ± 1.5 ^3^	ND

^1^ Inhibitory effects of samples against LPS-induced NO production in RAW264.7 cells. ^2^ Inhibition of cell viability during the periods of samples-treated RAW264.7 cells. ^3^ Positive control. ^4^ Not determined.

## Supplementary Materials

The supplementary materials are available online.
