# Supplementary material for: Semisynthesis, an Anti-Inflammatory Effect of Derivatives of 1β-Hydroxy Alantolactone from Inula britannica
Source: Molecules. 2017 Oct 27;22(11):1835. doi: 10.3390/molecules22111835 (PMC6150205; doi:10.3390/molecules22111835)
Supplement: Supplementary file 1 [file molecules-22-01835-s001.pdf]

Supplementary Material

Semisynthesis, an Anti-Inflammatory Effect of Derivatives of 1β-Hydroxy Alantolactone from *Inula britannica*

Lin Chen[1,2], Jian-Ping Zhang[3], Xin Liu[2], Jiang-Jiang Tang[4], Ping Xiang[5,*] and Xing-Ming Ma[1,*]

[1] Department of Immunology, Institute of Integrated Chinese and Western Medicine, School of Basic Medical Sciences, Lanzhou University, 730000, P.R. China; chenlinfree@126.com (L.C.)
[2] Department of Infectious Disease, the First Hospital of Lanzhou University, Lanzhou, 730000, P.R. China; liuxin1288@126.com (X.L.)
[3] Department of Pharmacy, the First Hospital of Lanzhou University, Lanzhou, 730000, P.R. China; Zhangjianping0306@163.com (J.-P.Z.)
[4] College of Chemistry & Pharmacy, Northwest A&F University, Yangling, 712100, P.R. China; tangjiang11@nwafu.edu.cn (J.-J.T.)
[5] College of Plant Protection, Northwest A&F University, Yangling, 712100, P.R. China;
* Correspondence: xiangp02@163.com (P.X.); maxm@lzu.edu.cn (X.-M.M.); Tel.: +86-298-703-4332 (P.X.)

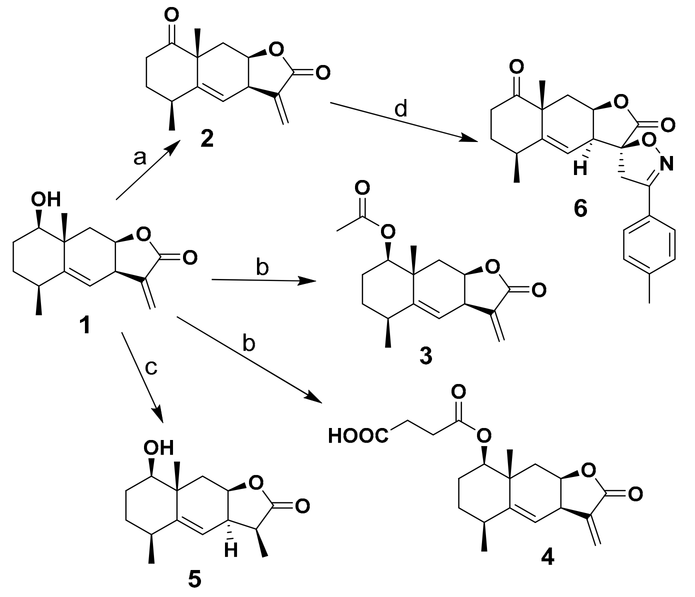

Scheme 1. Semisynthetic route of 1β-hydroxy alantolactone (**1**) derivatives.

The General Procedure for the Synthesis of Derivative 2

To a suspension of Dess–Martin periodinane (0.4 mmol) in anhydrous CH_2Cl_2 (1 mL), compound **1** (0.2 mmol) in anhydrous CH_2Cl_2 (1 mL) solution was added. The resulting solution was added to saturated aqueous $NaHCO_3$ and extracted with CH_2Cl_2. After removal of the solvent, the crude product was purified by silica gel chromatography (EtOAc/PE) to afford compound **2**.

Derivative 2: White powder. $[\alpha]_D^{30}$ = +190.5° (*c* 0.034 in $CHCl_3$); ^1H NMR (500 MHz, $CDCl_3$): δ 6.24 (d, *J* = 1.8 Hz, 1H, H-13a), 5.67 (d, *J* = 1.6 Hz, 1H, H-13b), 5.37 (d, *J* = 4.2 Hz, 1H, H-6), 4.84 (td, *J* = 6.6, 3.3 Hz, 1H, H-8), 3.56 (m, *J* = 9.6, 5.3 Hz, 1H, H-7), 2.74 (m, 1H, H-2a), 2.59 (m, 1H, H-4), 2.45 (dd, *J* = 15.0, 3.0 Hz, 1H, H-9a), 2.25 (m, 1H, H-2b), 1.95 (m, 1H, H-3a), 1.72 - 1.84 (m, 2H, H-3b, H-9b), 1.38 (s, 3H, H-14), 1.25 (d, *J* = 7.3 Hz, 3H, H-15); ^{13}C NMR (125 MHz, $CDCl_3$): δ 213.3 (C-1), 170.0 (C-12), 145.85 (C-5), 139.3 (C-11), 122.4 (C-6), 121.8 (C-13), 75.3 (C-8), 47.0 (C-10), 39.4 (C-7), 36.0 (C-9), 35.6 (C-4), 34.0(C-2), 28.7 (C-3), 28.7 (C-14), 23.0 (C-15); ESI-MS: *m/z* 515.34 $[2M+Na]^+$; HRMS (ESI): *m/z* calcd for $C_{15}H_{19}O_3$ $[M+H]^+$ 247.13287, found 247.13283.

The General Procedure for the Synthesis of Derivatives 3 and 4

To a suspension of anhydride (0.2 mmol), Et_3N (0.3 mmol) and DMAP (0.01 mmol) in anhydrous CH_2Cl_2 (1 mL) in an ice-bath stirred for 30 min, compound **1** or anhydrous CH_2Cl_2 (1 mL) solution was added. After completion of the reaction for 30 min at room temperature, ice water (2 mL) was added to the solvent and stirred for 20 min, then extracted with CH_2Cl_2, dried, and filtered. After removal of the solvent, the crude product was purified by silica gel chromatography (EtOAc/PE).

Derivative 3: White powder. $[\alpha]_D^{30}$ = +190.5° (*c* 0.036 in $CHCl_3$); ^{1}H NMR (500 MHz, $CDCl_3$): δ 6.20 (d, *J* = 1.6 Hz, 1H, H-13a), 5.63 (d, *J* = 1.3 Hz, 1H, H-13b), 5.25 (d, *J* = 3.8 Hz, 1H, H-6), 4.79 (m, 1H, H-8), 4.53 (dd, *J* = 11.7, 3.9 Hz, 1H, H-1), 3.56 (s, 1H, H-7), 2.45 (m, 1H, H-4), 2.27 (dd, *J* = 15.0, 2.7 Hz, 1H, H-9b), 2.07 (s, 3H, CH_3CO -1), 1.87 (m, 1H, H-2a), 1.72–1.63 (m, 2H, H-3a, H-2b), 1.60–1.50 (m, 2H, H-3b, H-9a), 1.24 (s, 3H, H-14), 1.10 (d, *J* = 7.6 Hz, 3H, H-15); ^{13}C NMR (125 MHz, $CDCl_3$): δ 170.9 (C-12), 170.2 (CH_3*C*O-1), 147.1 (C-5), 139.5 (C-11), 122.2 (C-13), 121.2 (C-6), 81.4 (C-1), 75.4 (C-8), 39.4 (C-7), 39.0 (C-9), 37.2 (C-10), 37.2 (C-4), 29.5 (C-3), 23.2 (*C*H_3CO-1), 22.7 (C-14), 22.4 (C-2), 21.3 (C-15); ESI-MS: *m/z* 271.5 $[M+Na]^+$; HRMS (ESI): *m/z* calcd for $C_{17}H_{23}O_4$ $[M+H]^+$ 291.15909, found 291.15909.

Derivative 4: Yellow powder. $[\alpha]_D^{30}$ = +63.34° (*c* 0.31 in $CHCl_3$); ^{1}H NMR (500 MHz, $CDCl_3$) δ 6.24 (d, *J* = 1.5 Hz, 1H, H-13a), 5.68 (d, *J* = 1.2 Hz, 1H, H-13b), 5.29 (d, *J* = 3.8 Hz, 1H, H-6), 4.85 – 4.80 (m, 1H, H-8), 4.60 (dd, *J* = 11.7, 3.9 Hz, 1H, H-1), 3.62 – 3.57 (m, 1H, H-7), 2.72 (m, 2H, H-18), 2.71 – 2.67 (m, 2H, H-17), 2.51 – 2.44 (m, 1H, H-4), 2.32 (m, 1H, H-9b), 1.92 (m, 1H, H-2b), 1.74 – 1.53 (m, 4H, H-2a, H-3, H-9a), 1.27 (s, 3H, H-14), 1.14 (d, *J* = 7.6 Hz, 3H, H-15); ^{13}C NMR (125 MHz, $CDCl_3$) δ 178.0 (C-19), 171.8 (C-16), 170.2 (C-12), 146.9 (C-5), 139.4 (C-11), 122.2 (C-13), 121.2 (C-6), 81.9 (C-1), 75.4 (C-8), 39.3 (C-9), 38.8 (C-7), 37.2 (C-10), 37.1 (C-4), 29.4 (C-3), 29.3 (C-17), 29.0 (C-18), 23.2 (C-2), 22.6 (C-14), 22.3 (C-15); HRMS (ESI): *m/z* calcd for $C_{19}H_{25}O_6$ $[M+H]^+$ 349.16456, found 349.16519.

The General Procedure for the Synthesis of Derivative 5

$NaBH_4$ (1.2 mmol) was added to a solution of 1β-hydroxy alantolactone (**1**) (0.3 mmol) in anhydrous THF (5 mL). The solution was stirred vigorously. The reaction was completed after ~2 h using TLC detection, and 1 M HCl (2 mL) solution was added to quench the reaction. The mixture was extracted with CH_2Cl_2, washed with brine, dried over anhydrous Na_2SO_4, and concentrated under reduced pressure. The residue was purified via silica column chromatography with EtOAc/PE (3:1)

Derivative 5: White powder. $[\alpha]_D^{30}$ = -15.7° (*c* 0.31 in MeOH); ^{1}H NMR (500 MHz, $CDCl_3$) δ 5.27 (d, *J* = 3.1 Hz, 1H, H-6), 4.77 (dt, *J* = 5.6, 2.7 Hz, 1H, H-8), 3.27 (dd, *J* = 11.7, 3.9 Hz, 1H, H-1), 3.00 (m, *J* = 8.7, 5.6, 3.2 Hz, 1H, H-7), 2.92–2.84 (m, 1H, H-11), 2.55 (dd, *J* = 14.8, 3.3 Hz, 1H, H-9b), 2.49–2.39 (m, 1H, H-4), 1.89–1.78 (m, 1H, H-2b), 1.67–1.58 (m, 2H, H-2a, H-9a), 1.54 (dt, *J* = 14.7, 2.8 Hz, 2H, H-3), 1.23 (d, *J* = 7.4 Hz, 3H, H-13), 1.22 (s, 3H, H-14), 1.12 (d, *J* = 7.6 Hz, 3H, H-15); ^{13}C NMR (125 MHz, $CDCl_3$) δ 179.0 (C-12), 149.4 (C-5), 117.6 (C-6), 80.8 (C-1), 76.3 (C-8), 40.5 (C-11), 39.7 (C-7), 38.6 (C-9), 38.5 (C-10), 38.2 (C-4), 29.8 (C-3), 26.1 (C-2), 23.1 (C-14), 21.9 (C-15), 10.6 (C-13); ESI-MS: *m/z* 522.92 $[2M+Na]^+$.

General Procedure for the Synthesis of Derivative 6

To a solution of newly-made *p*-methylbenzaldoxime chloride (0.105 mmol) and **2** (0.1 mmol) in CH_2Cl_2 (3 mL), Et_3N (0.125 mmol) at 0 °C was added. The resulting mixture was stirred at room temperature for 12 h. The solvent was evaporated in vacuo and the residue was purified via silica column chromatography with (EtOAc/PE) as eluent to provide compounds **6**.

Derivative 6: White powder. $[\alpha]_D^{30}$ = +208.7° (*c* 0.28 in MeOH); ^{1}H NMR (500 MHz, $CDCl_3$): δ 7.59 (d, *J* = 8.2 Hz, 2H, H-19, H-19′), 7.23 (d, *J* = 7.9 Hz, 2H, H-18, H-18′), 5.34 (d, *J* = 3.4 Hz, 1H, H-6), 5.19 (m, *J* = 5.3, 2.8 Hz, 1H, H-8), 3.68 (d, *J* = 17.0 Hz, 1H, H-13a), 3.49 (d, *J* = 17.0 Hz, 1H, H-13b), 3.10-3.06 (m, 1H, H-7), 2.76–2.66 (m, 2H, H-2a, H-4), 2.52 (dd, *J* = 15.6, 3.6 Hz, 1H, H-9a),2.39 (s, 3H, H-20-Me), 2.24 (m, *J* = 16.2, 9.4, 4.9 Hz, 1H, H-2b), 2.02–1.94 (m, 1H, H-3a), 1.90 (dd, *J* = 15.6, 2.6 Hz, 1H, H-9b), 1.82–1.75 (m, 1H, H-3b), 1.45-1.41 (m, 3H, H-14), 1.30 (d, *J* = 7.3 Hz, 3H, H-15); ^{13}C NMR (125 MHz, $CDCl_3$): δ 212.5 (C-1), 173.0 (C-12), 156.4 (C-16),

149.9 (C-5), 141.2 (C-20), 129.6 (C-17), 126.9 (C-19, C-19′), 125.6 (C-18, C-18′), 116.2 (C-6), 89.6 (C-11), 76.4 (C-8), 47.2 (C-10), 43.0 (C-7), 37.0 (C-13), 36.6 (C-4), 35.5 (C-2), 33.8 (C-9), 28.2 (C-3), 28.1 (C-14), 23.7 (C-15), 21.5 (C-20-Me); HRMS (ESI): *m/z* calcd for $C_{23}H_{26}NO_4$ $[M+H]^+$ 380.18563, found 380.18564; calcd for $C_{46}H_{51}N_2O_8$ $[2M+H]^+$ 759.36399, found 759.36407; HPLC: t_R = 39.0 min, purity = 95.1% @ 270 nm, 0-100% methanol in water for 50 min.

Spectral data for derivatives **2**–**6**:

NMR Spectra and HRESI-MS

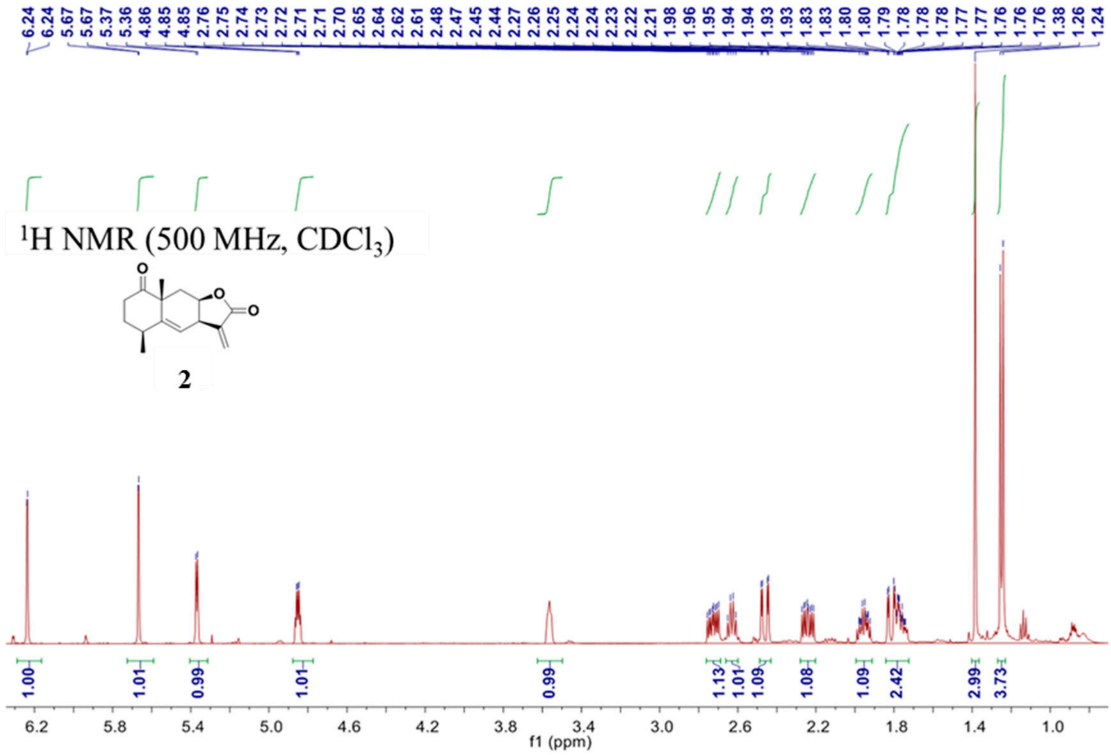

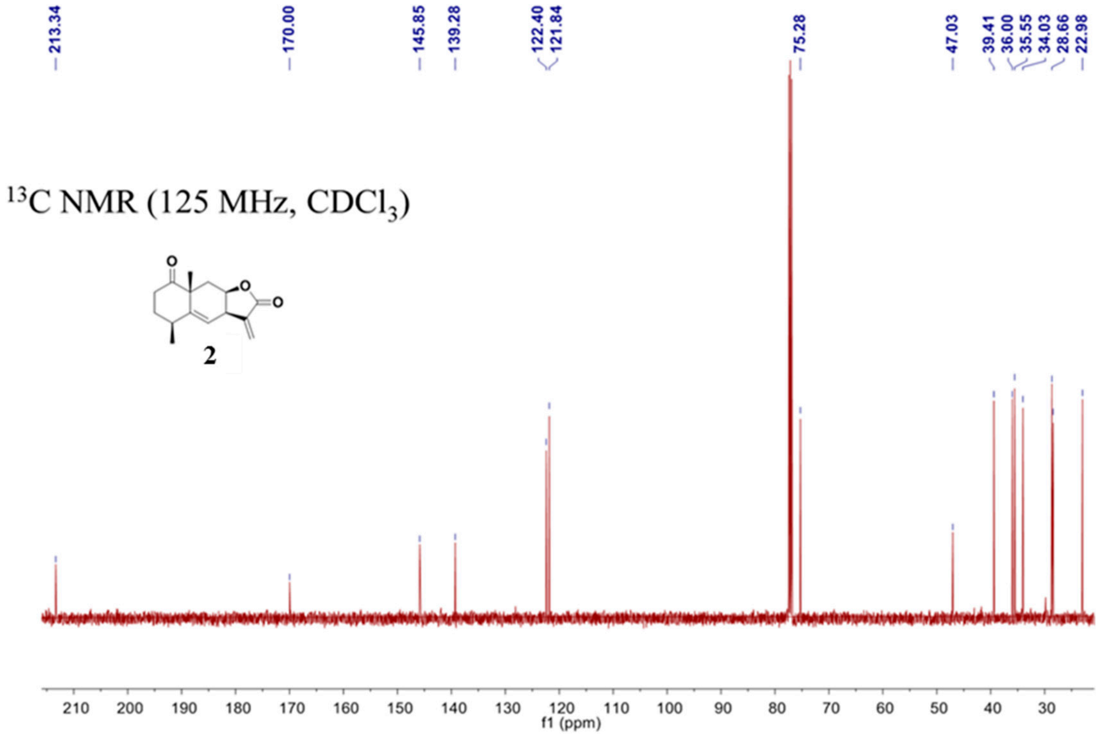

HRMS (ESI) m/z calcd for $C_{15}H_{19}O_3$ $(M+H)^+$ 247.13287, found 247.13283.

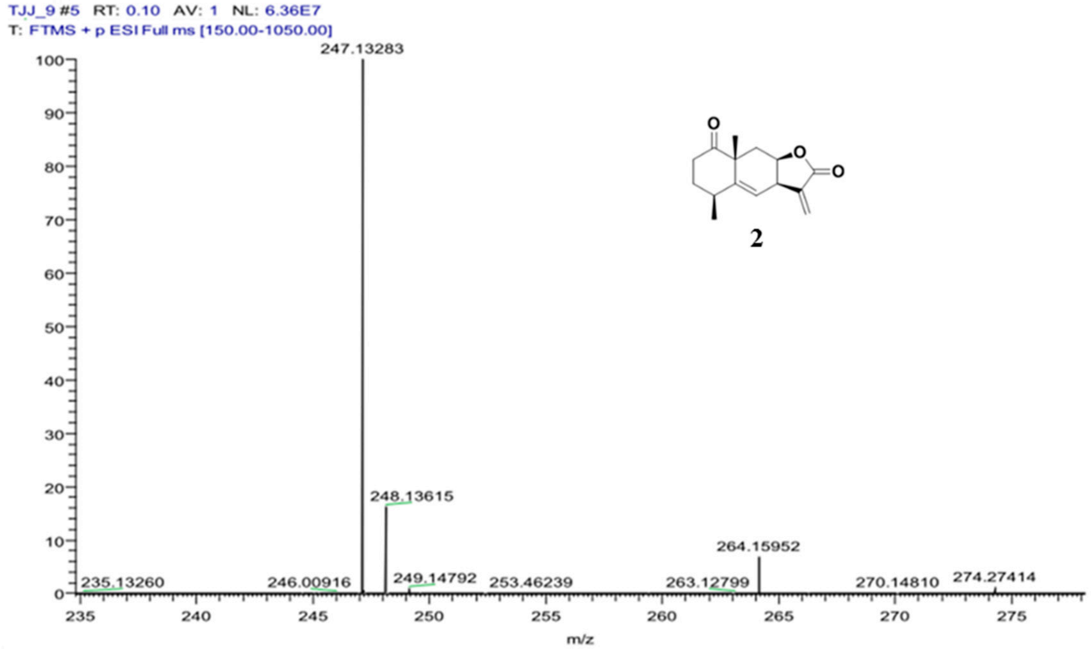

^{1}H NMR (500 MHz, CDCl$_3$)

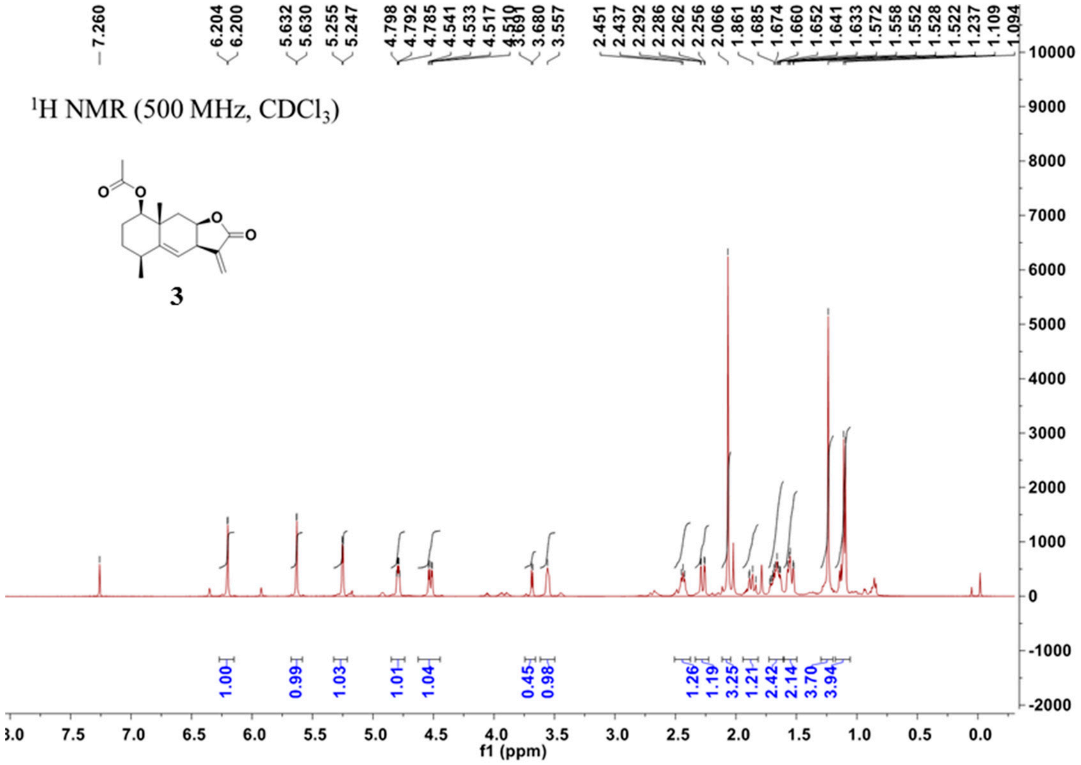

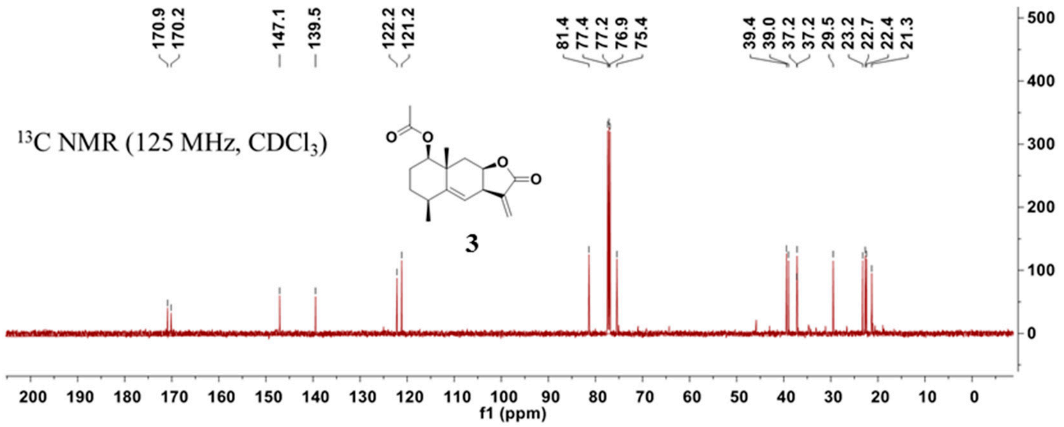

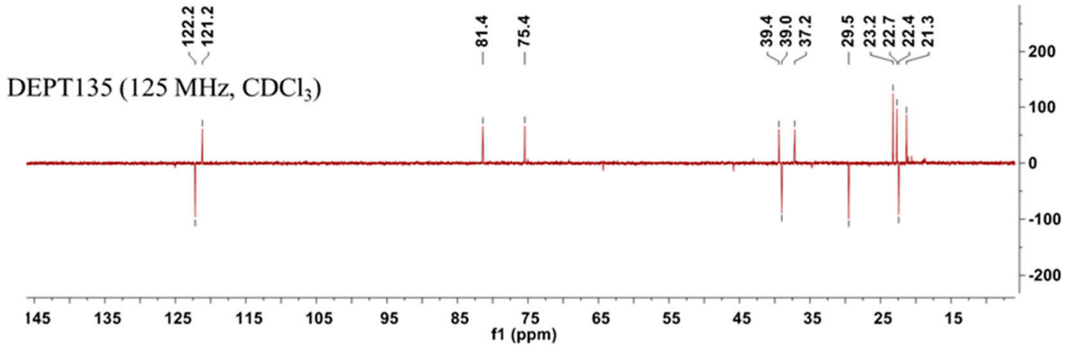

HRMS (ESI) m/z calcd for $C_{17}H_{23}O_4$ $(M+H)^+$ 291.15909, found 291.15909.

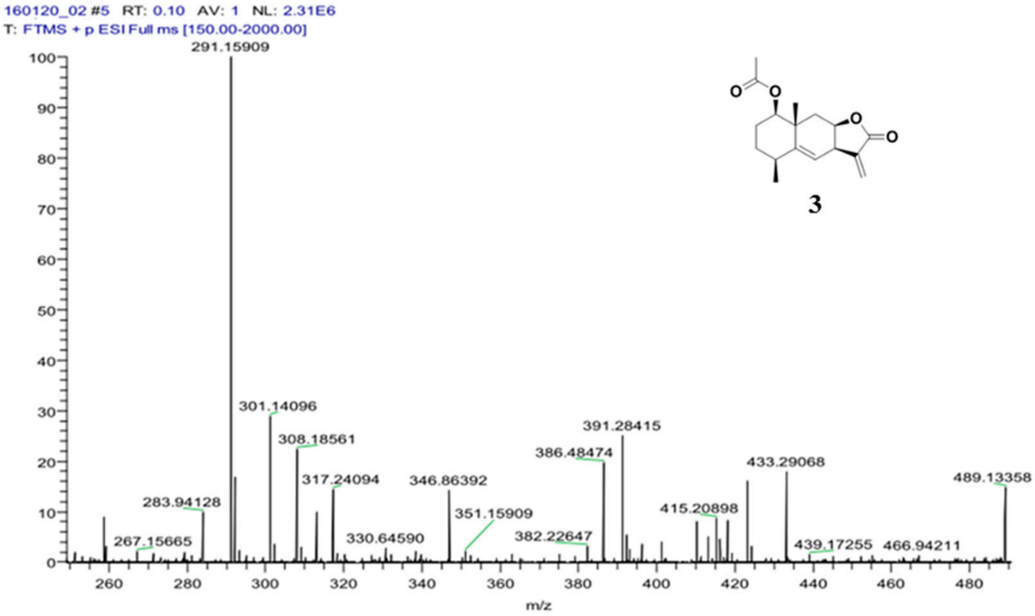

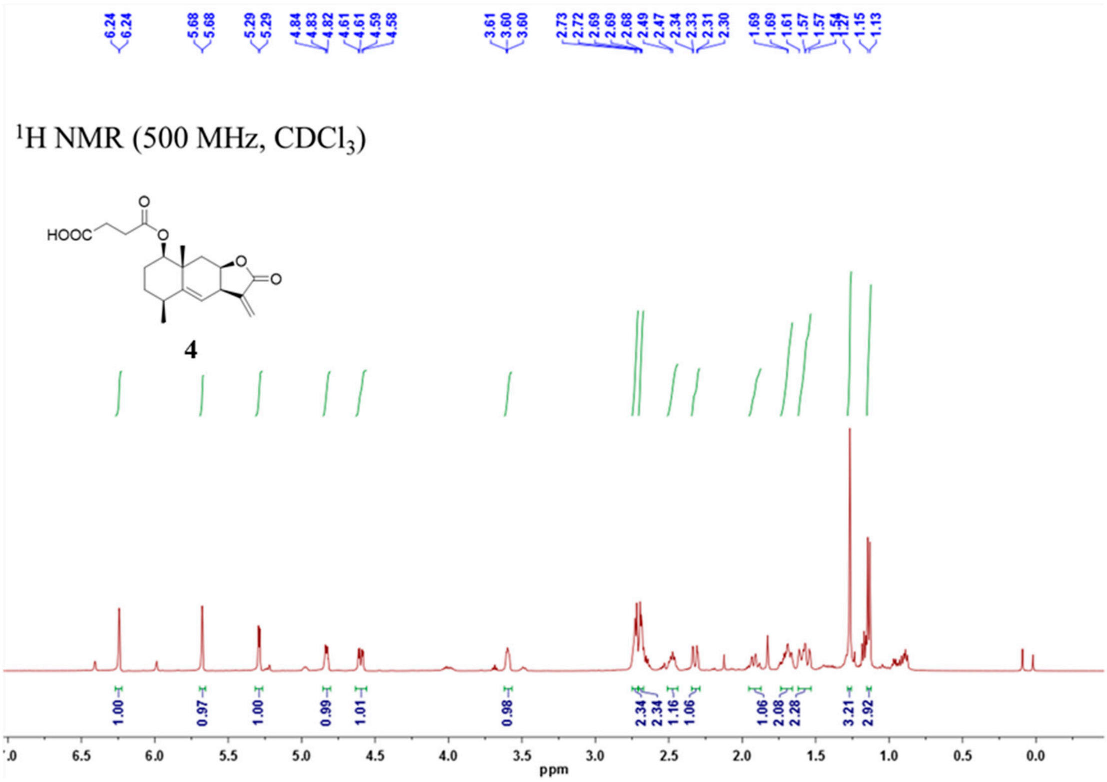

^{1}H NMR (500 MHz, $CDCl_3$)
HOOC
4
ppm

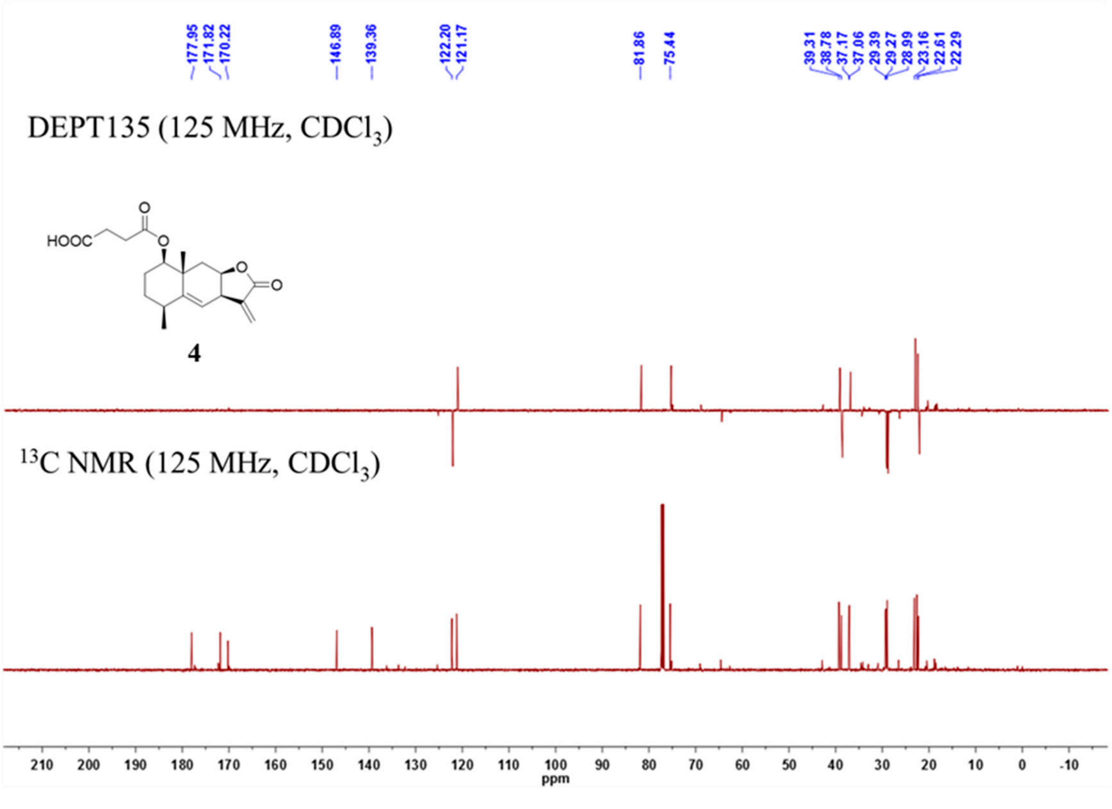

DEPT135 (125 MHz, $CDCl_3$)
HOOC
4
^{13}C NMR (125 MHz, $CDCl_3$)
ppm

HRMS (ESI) *m/z* calcd for $C_{19}H_{25}O_6$ $(M+H)^+$ 349.16456, found 349.16519.

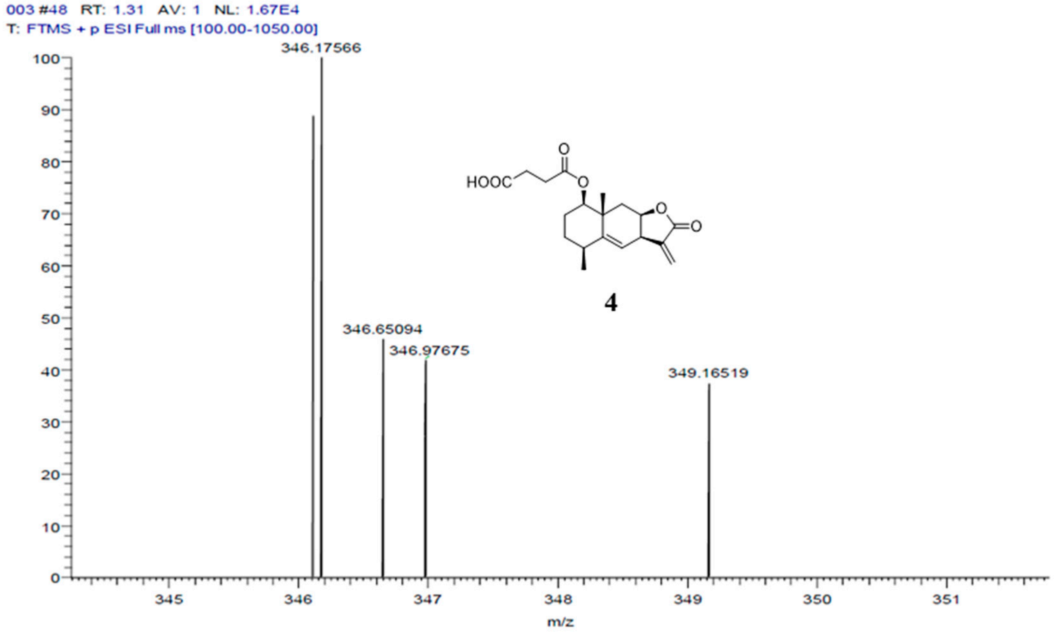

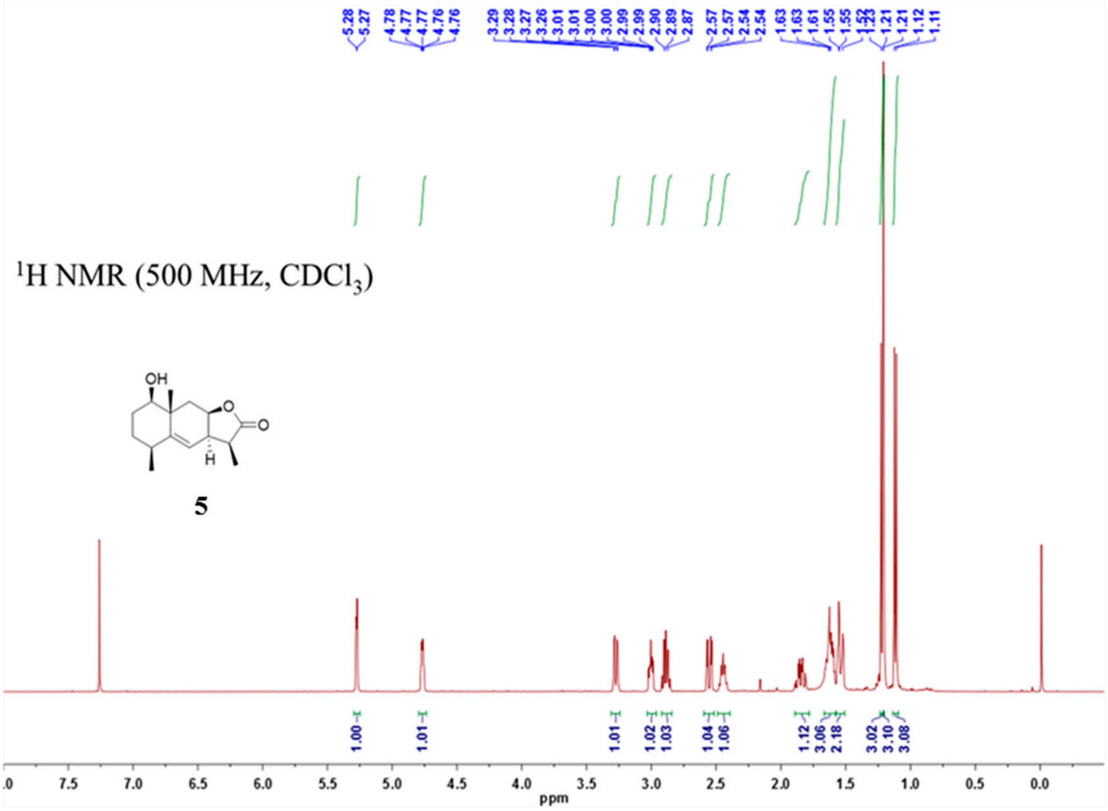

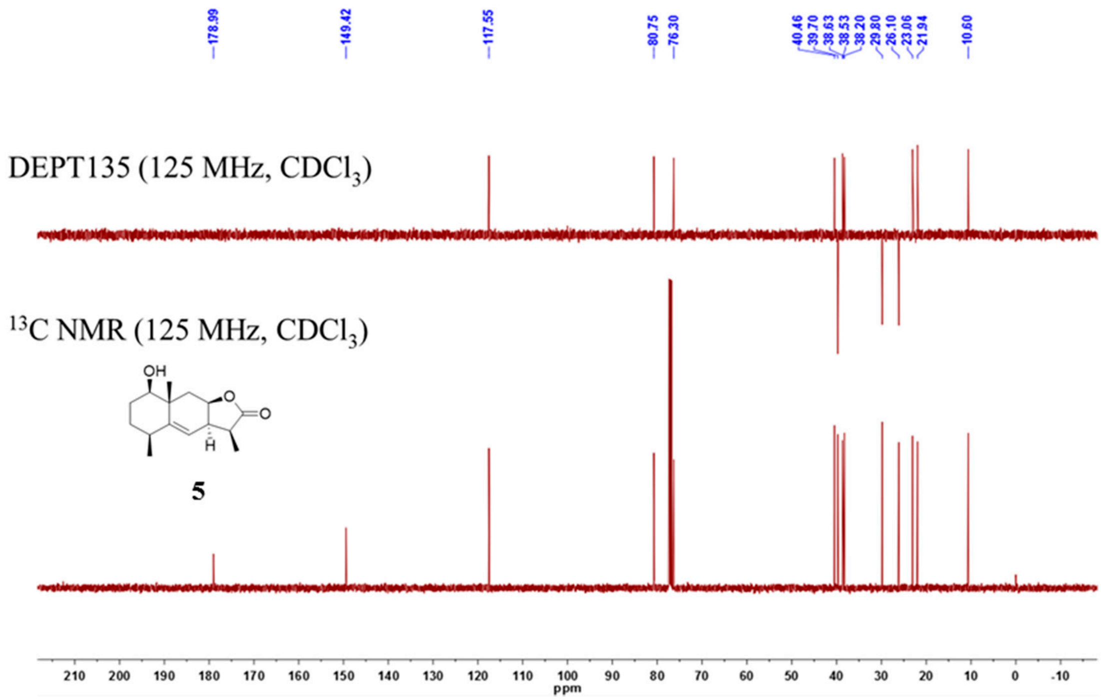
178.99
149.42
117.55
80.75
76.30
40.46
39.70
38.63
38.53
38.20
29.80
26.10
23.06
21.94
10.60
DEPT135 (125 MHz, CDCl3)
13C NMR (125 MHz, CDCl3)
OH
O
O
H
5
210 200 190 180 170 160 150 140 130 120 110 100 90 80 70 60 50 40 30 20 10 0 -10
ppm

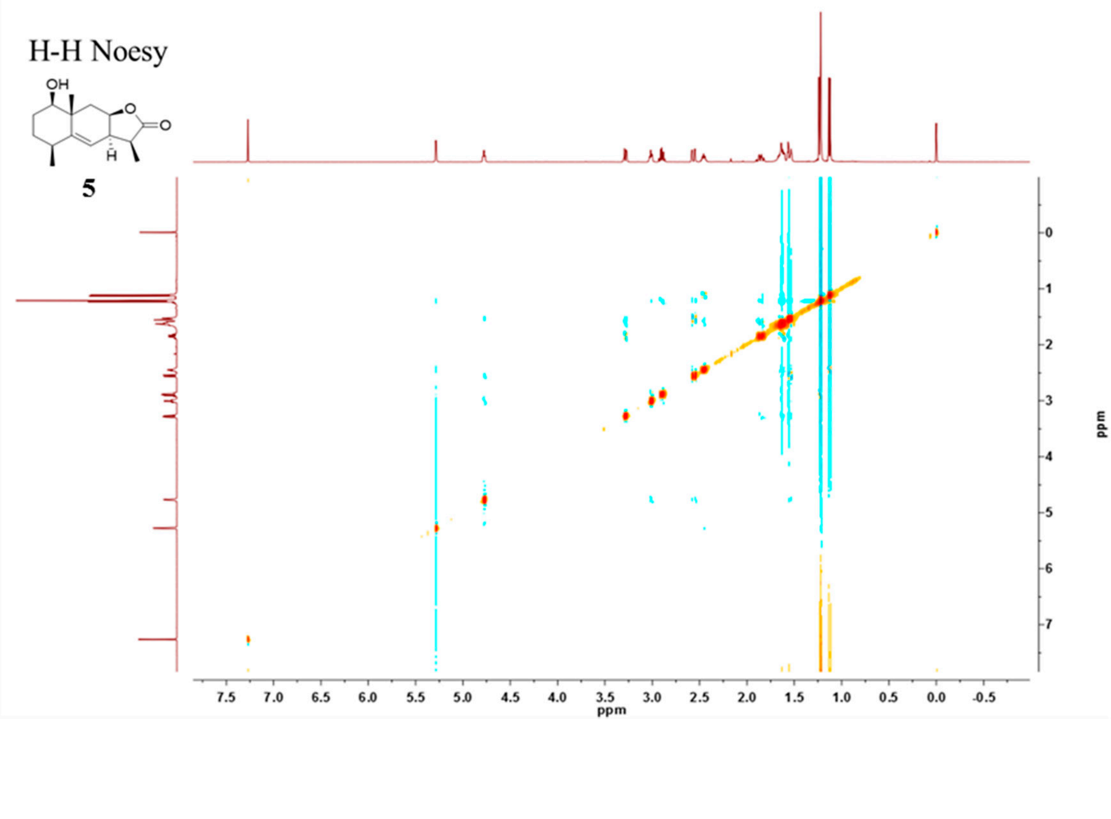
H-H Noesy
OH
O
O
H
5
7.5 7.0 6.5 6.0 5.5 5.0 4.5 4.0 3.5 3.0 2.5 2.0 1.5 1.0 0.5 0.0 -0.5
ppm
0 1 2 3 4 5 6 7
ppm

HRMS (ESI) *m/z* calcd for $C_{15}H_{23}O_3$ $(M+H)^+$ 251.16417, found 251.16432.

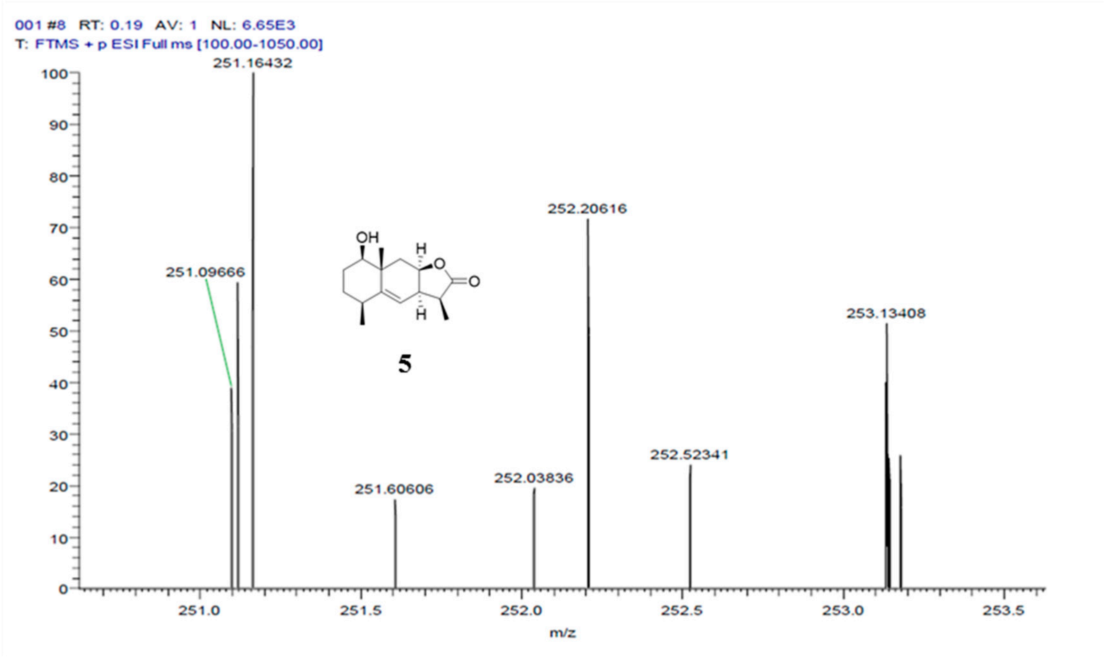

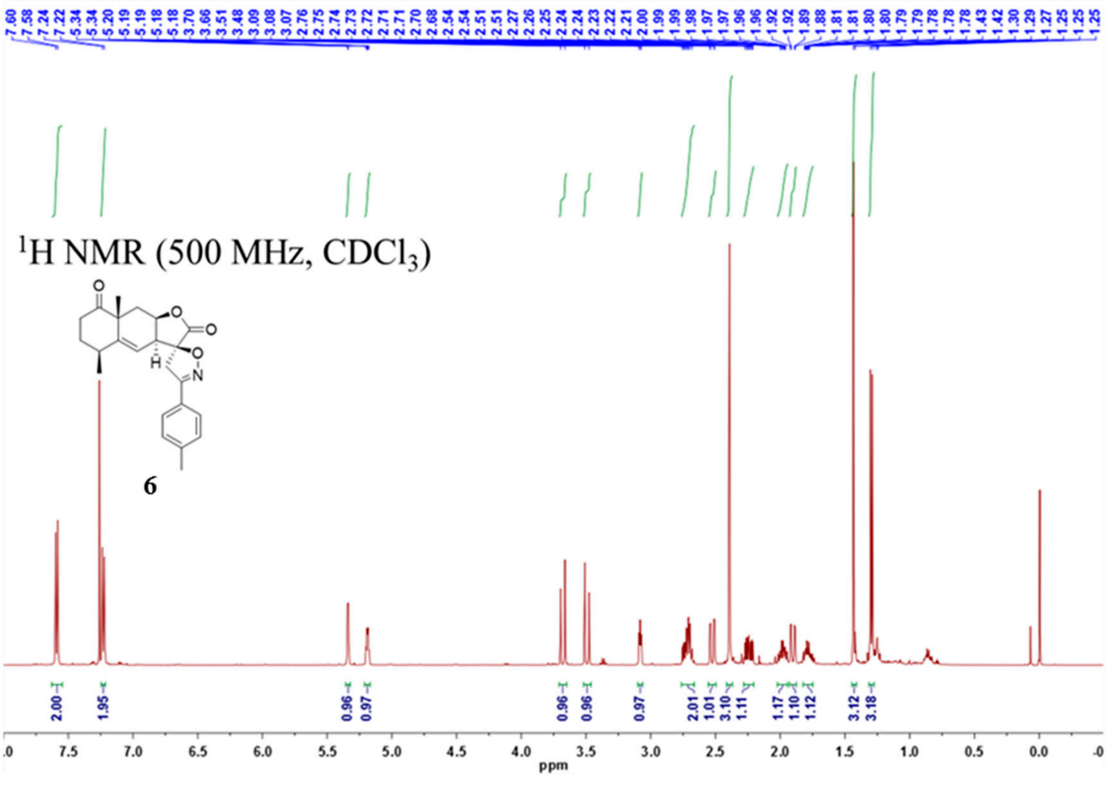

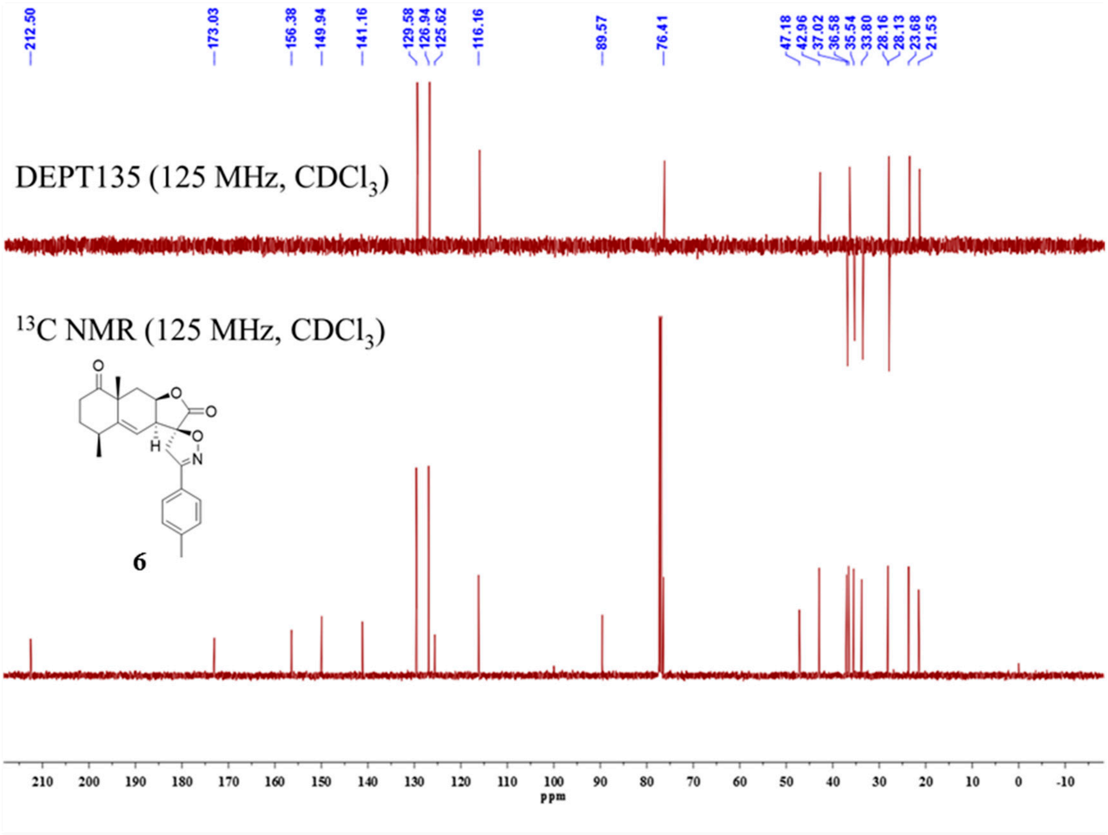

HRMS (ESI) *m/z* calcd for $C_{23}H_{26}NO_4^+$(M+H)$^+$ 380.18563, found 380.18564; calcd for $C_{46}H_{51}N_2O_8^+$(2M+H)$^+$ 759.36399, found 759.36407.

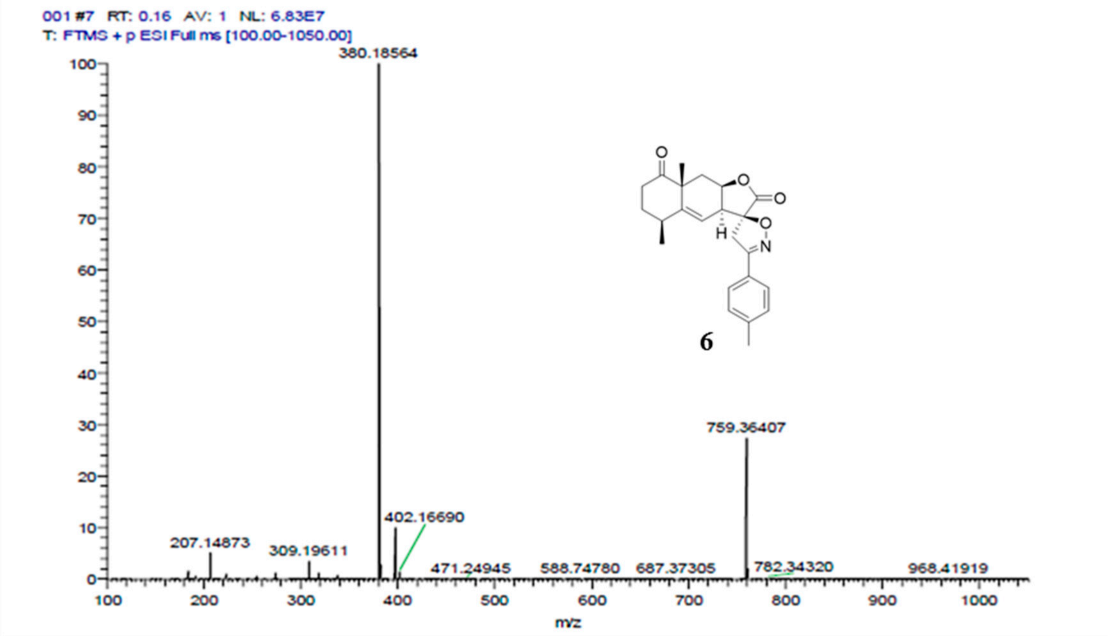

HPLC purity determination of **1–6**

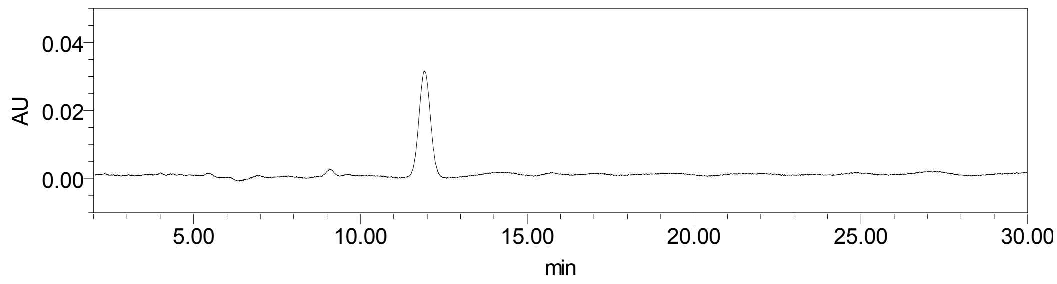

1: Purity 96.1%, t_R = 11.9 min at 215 nm by RP-HPLC using 50% methanol in water.

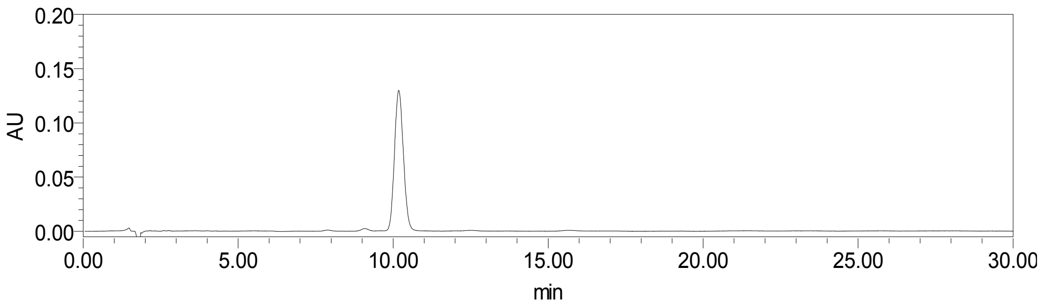

2: Purity 97.0%, t_R = 10.2 min at 230 nm by RP-HPLC using 50% methanol in water.

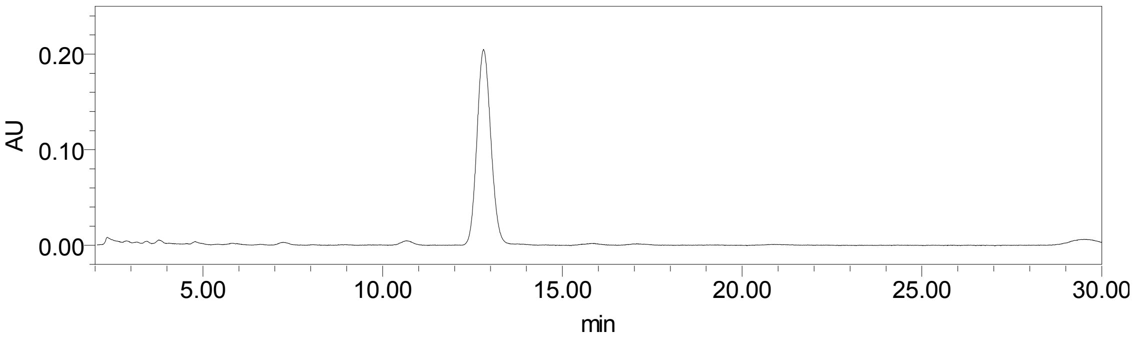

3: Purity 95.2%, t_R = 12.8 min at 210 nm by RP-HPLC using 60% methanol in water.

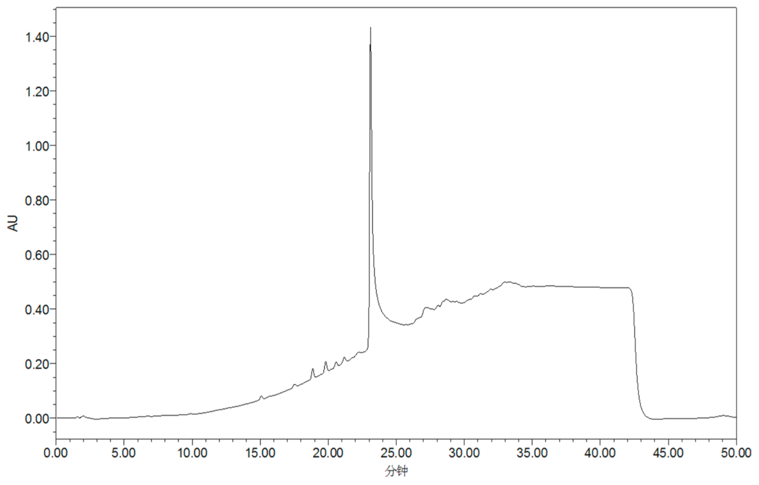

4: Purity 95.4%, t_R = 23.1 min at 210 nm by RP-HPLC using a 50 min gradient from 0% to 100% of methanol in water.

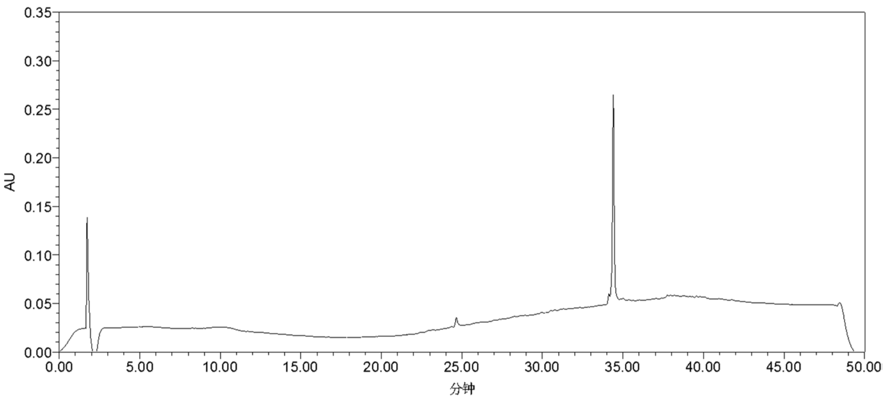

5: Purity 98.5%, t_R = 34.4 min at 210 nm by RP-HPLC using 50 min gradient from 0% to 100% of methanol in water.

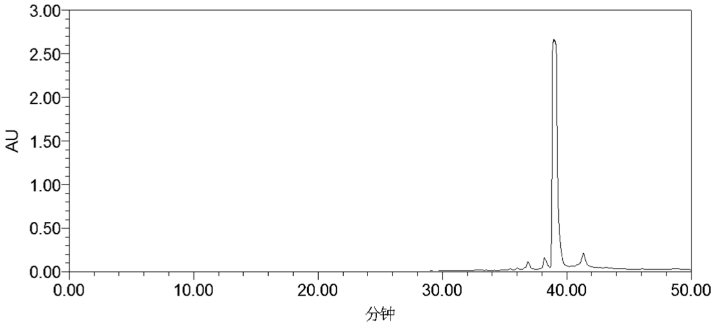

6: Purity 95.1%, t_R = 39.0 min at 270 nm by RP-HPLC using 50 min gradient from 0% to 100% of methanol in water.
